# Supplementary material for: An integrative transcriptomic atlas of organogenesis in human embryos
Source: eLife. 2016 Aug 24;5:e15657. doi: 10.7554/eLife.15657 (PMC4996651; doi:10.7554/eLife.15657)
Supplement: Supplementary file 5. — High resolution version of Figure 1—figure supplement 1 where each individual transcription factor is text searchable. (Please note that this opens at 25 x 200 inches / 64 x 508 cm). DOI: http://dx.doi.org/10.7554/eLife.15657.018 [file elife-15657-supp5.pdf]

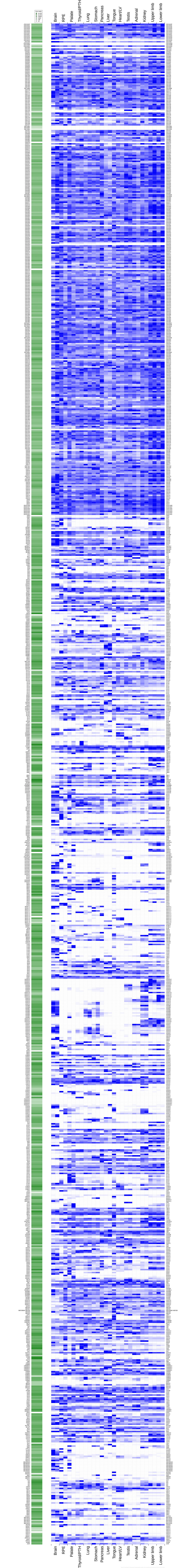
Brain
RPE
Palate
Thyroid/PTH
Lung
Stomach
Pancreas
Liver
Tongue
Heart/LV
Testis
Adrenal
Kidney
Upper limb
Lower limb
